# Validation of a wireless dry electrode system for electroencephalography

**DOI:** 10.1186/s12984-015-0089-2

**Published:** 2015-10-31

**Authors:** Sarah N Wyckoff, Leslie H Sherlin, Noel Larson Ford, Dale Dalke

**Affiliations:** SenseLabs, Mesa, Arizona, Atascadero, CA USA; Department of Psychology, Northern Arizona University, Flagstaff, AZ USA; Department of Mind-Body Medicine, Southwest College of Naturopathic Medicine, Tempe, AZ USA

**Keywords:** Dry electrode, Electroencephalography, Signal generator, Signal quality, Validity, Wireless EEG

## Abstract

**Background:**

Electroencephalography (EEG) is a widely used neuroimaging technique with applications in healthcare, research, assessment, treatment, and neurorehabilitation. Conventional EEG systems require extensive setup time, expensive equipment, and expertise to utilize and therefore are often limited to clinical or laboratory settings. Technological advancements have made it possible to develop wireless EEG systems with dry electrodes to reduce many of these barriers. However, due to the lack of homogeneity in hardware, electrode evaluation, and methodological procedures the clinical acceptance of these systems has been limited.

**Methods:**

In this investigation the validity of a wireless dry electrode system compared to a conventional wet electrode system was assessed, while addressing methodological limitations. In Experiment 1, the signal output of both EEG systems was examined at Fz, C3, Cz, C4, and Pz using a conductive head model and generated test signals at 2.5 Hz, 10 Hz, and 39 Hz. In Experiment 2, two-minutes of eyes-closed and eyes-open EEG data was recorded simultaneously with both devices from the adjacent electrode sites in a sample of healthy adults.

**Results:**

Between group effects and frequency*device and electrode*device interactions were assessed using a mixed ANOVA for the simulated and in vivo signal output, producing no significant effects . Bivariate correlation coefficients were calculated to assess the relationship between electrode pairs during the simultaneous in vivo recordings, indicating a significant positive relationship (all *p*'s < .05) and larger correlation coefficients (*r* > *±* 0.5) between the dry and wet electrode signal amplitude were observed for theta, alpha, beta 1, beta 2, beta 3, and gamma in both the eyes-closed and eyes-open conditions.

**Conclusions:**

This report demonstrates preliminary but compelling evidence that EEG data recorded from the wireless dry electrode system is comparable to data recorded from a conventional system. Small correlation values in delta activity were discussed in relation to minor differences in hardware filter settings, variation in electrode placement, and participant artifacts observer during the simultaneous EEG recordings. Study limitations and impact of this research on neurorehabilitation were discussed.

## Background

Non-invasive electroencephalography (EEG) is a neuroimaging technique that measures cortical electrical activity of the brain with applications in healthcare, research, assessment, treatment, and neurorehabilitation. Digital (conventional) EEG systems are considered the established guideline for clinical EEG acquisition; recording voltage fluctuations using wired electrodes, digital amplifiers, and a direct connection to a laptop or desktop computer for data storage and analysis [1]. In clinical settings, a registered electroneurodiagnostic technologist and clinical electroencephalographer facilitate the acquisition and interpretation of clinical EEG recordings, while trained EEG technicians working under the supervision of a qualified electroencephalographer may facilitate data collection and analysis of EEG recordings from research and non-clinical populations in a laboratory setting [2]. The standard procedure for data collection requires accurate identification of recording sites (International 10–20 system), electrode site preparation with abrasive cleaners, electrode application/fixation (single lead with conductive paste or electrode cap system with injected conductive gel), and proper ground and reference electrode placement [3]. The cumbersome nature of conventional EEG systems and the need for assistive application make it difficult to conduct research outside of controlled clinical and laboratory settings, limiting in vivo and ambulatory research opportunities. Additionally, these limitations, as well as the high cost of conventional systems, create barriers for providers and individuals interested in utilizing EEG-based applications such as neuropsychological assessment, neurofeedback, or brain-computer interface for restorative or assistive neurorehabilitation or treatment monitoring.

In recent years, wireless technology and advancements in conductive materials have led to the development of several wireless EEG dry electrode systems for research and commercial use. Several validation studies have directly compared the signal output of dry and wet (pasted/gelled) electrode systems (review, see [4–6]). However this body of research has been criticized due to the lack of homogeneity in hardware and electrode evaluation procedures and statistical methodology. In a recent review, Gargiulo and colleagues [5] highlight several problems associated with current validation procedures; recommending researchers provide a comparative assessment of the proposed device with a reference device, thorough quantitative measurement and characterization of the electrical circuit of study devices, qualitative evaluations of physiological signals, report of compliance with technical standards, and long-term monitoring and multicenter studies to facilitate clinical acceptance. In their review of dry electrode validation research, Lopez-Gordo and colleagues [6] emphasize that heterogeneity in evaluation procedures limit the comparison of results between investigations and suggest mandatory reporting of the following study related characteristics: mechanical, electrical, evaluation, and usability.

The current investigation evaluates the validity of a wireless dry electrode system compared to a conventional wet electrode system, while addressing methodological limitations and complying with recommended reporting practices to standardize EEG system validation research. In a series of experiments, quantitative and qualitative aspects of the study related hardware and electrode performance were evaluated using simulated and in vivo recording techniques.

## Methods

### Ethics

All participants were provided written informed consent in accordance with the ethical conditions set forth as part of a larger data collection study overseen by the Western IRB (#20141246). Participants provided written consent to allow their data to be stored in a large database, de-identified, and published.

### Study devices

**Fig. 1:**
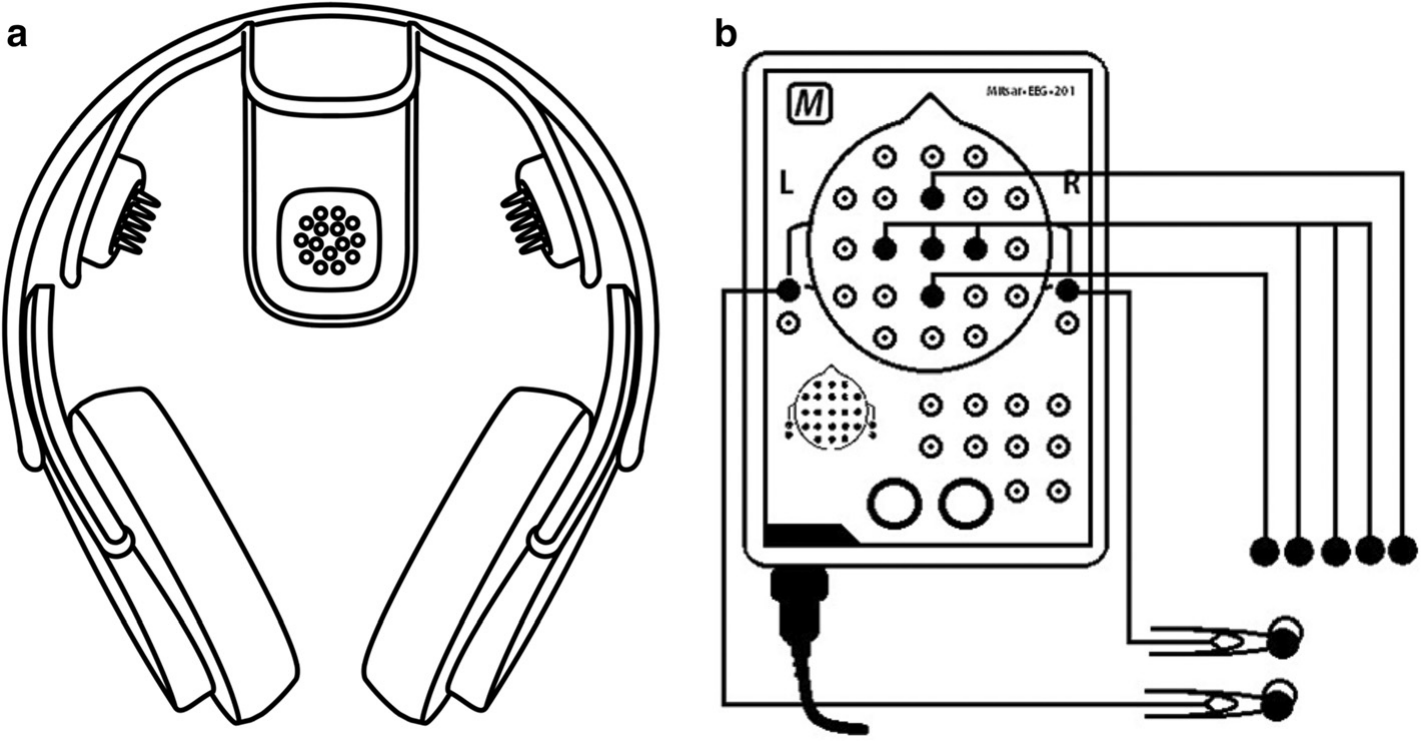
Study devices . Graphic representation of **a** Versus wireless electrode system with carbon-silicon dry electrodes and **b** Mitsar 201 amplifier with DIN style gold wet electrodes

**Fig. 2:**
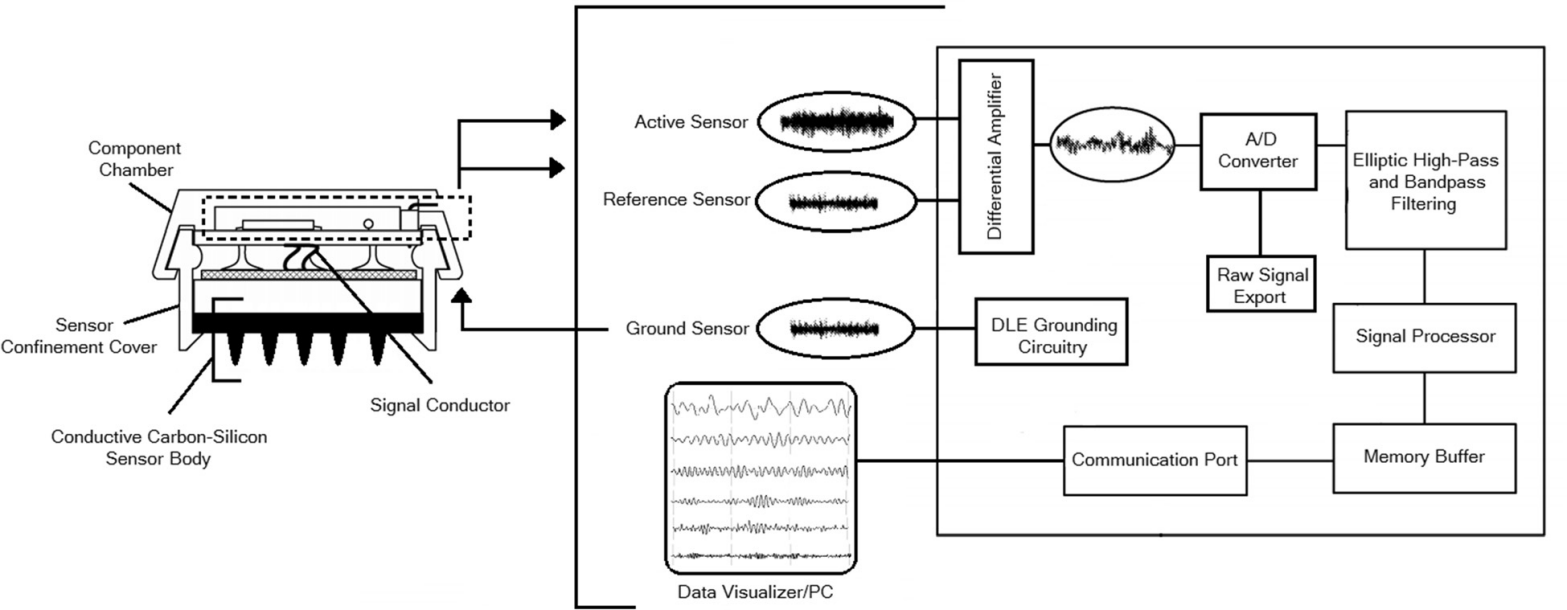
Diagram of the Versus headset dry electrode system

For this investigation, study devices included the Versus wireless dry electrode system (SenseLabs, Mesa, AZ & Atascadero, CA, USA) and the Mitsar-201 conventional wet electrode system (Mitsar Ltd, St. Petersburg, Russia). Figure 1 provides a visual representation of the study devices. Table 1 provides the system specifications of the study devices. The Versus wireless headset with EEG Stream software features five embedded 15-prong carbon-silicon dry electrodes at the following International 10–20 locations (Fz, C3, Cz, C4, Pz) with an integrated double-sided reference-ground earclip. Figure 2 provides a diagram of the Versus headset dry electrode and electrical circuit. The Mitsar-201 was selected as the reference device in the investigation, as it is a widely used, laboratory-based, EEG amplifier with 510 K (K143233) approval from the FDA. The Mitsar-201 amplifier with WinEEG software allows for the input of 19 EEG, 2 reference (A1 and A2), and 1 ground electrode using individual DIN style electrodes or an electrode cap with a serial port connector. For this investigation, single lead 9 mm flat DIN style gold electrodes were used for comparative testing with the conventional wet electrode system. Both study devices were in compliance with the guidelines set forth by the American Clinical Neurophysiology Society for the technical requirements for recording of EEG [7, 8] and other published technical standards [9]. The wireless technology utilized by the Versus headset is equivalent to that of a cell phone or wireless cellular headset and compliant with and eligible for the "low power exclusion" under the COMAR [10] standards for exposure to radio frequency devices.

**Table 1:** System specifications for study devices

Variable	Versus Headset	Mitsar - 201
Platforms	Windows XP, 7, 8	Window XP, 7, 8
Electrodes	Integrated - Carbon-Silicon	Single lead - 9 mm flat DIN gold
Fixation	Headset, direct contact, paste-free	Direct contact, conductive paste
Amplifier Power Supply	Rechargeable Li-poly (micro-USB)	Direct PC connection (USB)
Amplifier Current Absorption	60 mA	100 mA
Continuous Operation Time	5 h	8 h
Channels	5 (Fz, C3, Cz, C4, Pz)	21
Reference	1 (A1)	2 (A1 + A2)
Data Transmission	Bluetooth 2.1	USB
RF Frequency	2.4-2.48 GHz	N/A
RF Range	10 m	N/A
UART Baud Rate	115,200	460,800
Hardware Bandwidth	1-1 k Hz	0.16 - 70 Hz
Filter Type	Elliptical	IIR
Filter Order	Multiple	Multiple
Input Voltage Range	0.4 – 820 μV	1.5 - 5000 μV
Input Impedance	100 MΩ	Not < 200 MΩ
Electrode Impedance	~ 100 Ω	< 5 kΩ
Input Referred Noise	< 0.4 μVpp	< 1.5 μVpp
ADC Resolutions	12-bit	16-bit
Sampling rate	250/1280 sample/s	250/500 sample/s
Weight	344 g	0.9 kg

### Description of experiments

In experiment 1, study devices were compared using a signal generator to apply multiple test signals to a conductive head model with wet and dry electrodes attached at Fz, C3, Cz, C4, and Pz, with the Versus reference/ground electrode attached to the left ear (A1), and the Mitsar references and ground electrodes connected to the left and right ear and forehead, A1, A2, and FPz - respectively. In a series of 5-min serial EEG recordings, a 2.5 Hz, 10 Hz, and 39 Hz test signal was applied under a low and high resistance condition. The low resistance condition simulated proper electrode connection and the high resistance condition simulated poor electrode connection.

**Fig. 3:**
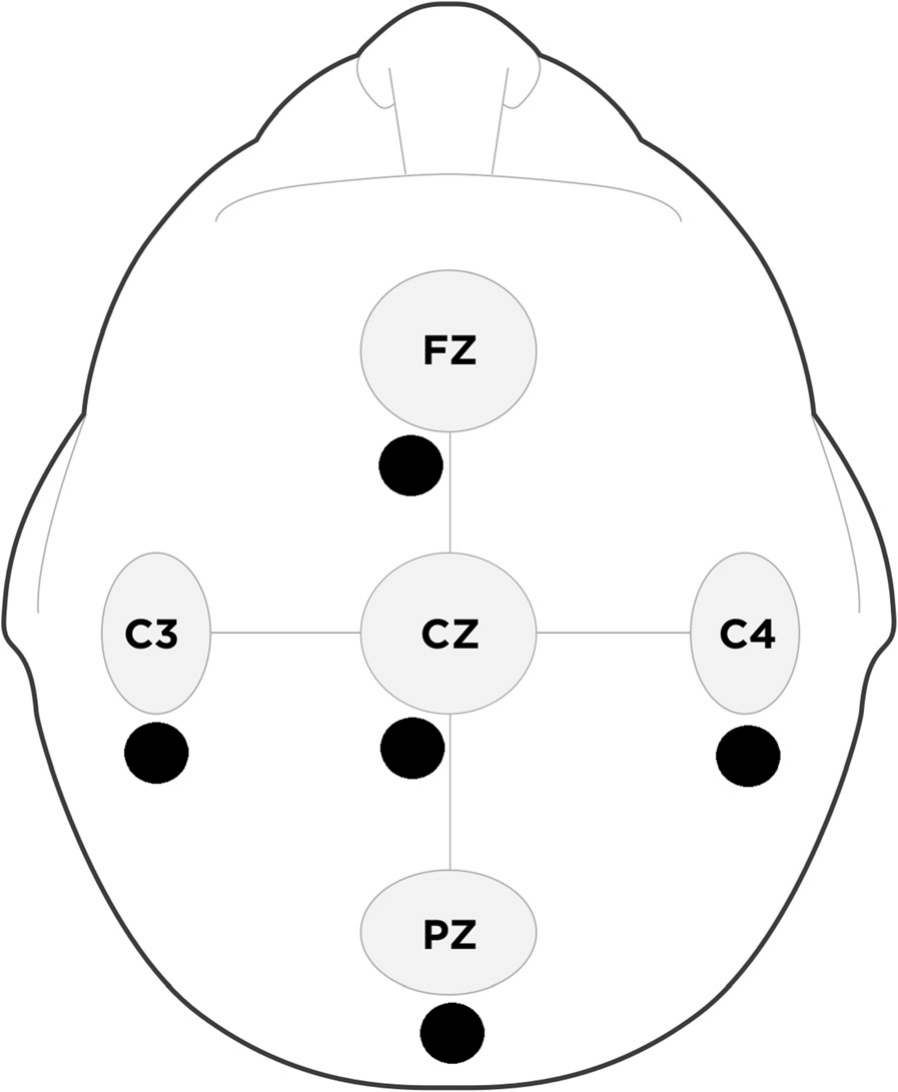
Signal validation electrode sites, in accordance with the International 10–20 System for Electrode Placement. The grey adjoined circles [larger] indicate the sites for the Versus headset dry electrodes and the black solid circles [smaller] indicate the sites for the Mitsar wet electrodes

In experiment 2, study devices were compared using simultaneous in vivo recordings from a healthy adult sample. Participants were seated comfortably in a reclining chair located in a private, climate controlled, light and sound attenuated recording room and were requested to remain relaxed and keep their eyes focused in a fixed direction throughout the recording to minimize electromyography and electrooculargraphic artifacts. Study tasks included a five-minute eyes-closed resting-state condition and a five-minute eyes-open resting-state condition. Due to the fixed electrode positioning of the Versus headset, dry electrodes were located at Fz, C3, Cz, C4, and Pz with the reference and ground electrodes fixed to the left ear-lobe (A1). Under the supervision of the EEG technician, participants were instructed to place the headset on their head and gently rock the device back and forth to allow the flexible carbon-silicon electrode protuberances to make contact with their scalp. Participants then clipped the reference/ground electrode to their left ear. Application of the Versus headset took approximately 2-min following the demonstration and required minimal assistance from the EEG technician. Single lead wet electrodes were fixed to the scalp using conductive paste by the EEG technician. The wet electrodes were placed posterior to the C3, Pz, and C4 dry electrodes, and to slightly to the left posterior of the Fz and Cz dry electrode with the references and ground electrodes connected to the left and right ear and forehead, A1, A2, and FPz - respectively. The site preparation and application of the 8 single lead electrodes took approximately 10-min. The placements of dry and wet electrodes for the simultaneous recordings are depicted in Fig. 3.

### Signal processing

For both experiment 1 and 2, data collected using the Versus headset was exported in ASCII (.txt) format and data collected using the wet electrode system was re-referenced to A1 and exported in EDF format. All data files were imported and processed using the Brain Vision Analyzer software (version 1.05, Brain Product GmbH, Germany). Each record was down-sampled to 128sps, bandpass filtered from 1.5-45 Hz, and synchronization markers were manually applied based on participant artifacts prompted at the onset of each task condition. Two minutes of continuous non-artifacted data from each condition (signal generator test, eyes-closed, and eyes-open) were segmented into 1 s epochs and subjected to Fast Fourier Transform (FFT) analysis (full spectrum, power, 0.5 Hz resolution, no windowing). Mean amplitude values (uV) for delta (1.5-3.5 Hz), theta (4–7.5 Hz), alpha (8–12 Hz), beta 1 (13–16 Hz), beta 2 (13–21 Hz), beta 3 (21–32 Hz), and gamma (35–45 Hz) frequency bands at each electrode site (Fz, C3, Cz, C4, and Pz) was exported for statistical analysis using IBM SPSS Statistics (Version 20).

### Statistical analysis

The following hypothesis was tested in experiment 1. Hypothesis 1: mean amplitude values for delta, alpha, and gamma frequency bands at Fz, C3, Cz, C4, and Pz *will not* be significantly different between the Versus dry electrode system and the Mitsar wet electrode system during the signal generator testing protocol. The following hypotheses were tested in experiment 2. Hypothesis 2: mean amplitude values for delta, theta, alpha, beta 1, beta 2, beta3, and gamma frequency bands at Fz, C3, Cz, C4, and Pz *will not* be significantly between the Versus dry electrode system and the Mitsar wet electrode system during the eyes-closed and eyes-open in vivo participant testing protocols. Hypothesis 3: mean amplitude values for delta, theta, alpha, beta 1, beta 2, beta3, and gamma frequency bands *will* be significantly correlated between the Versus dry electrode system and the Mitsar wet electrode system during the eyes-closed and eyes-open in vivo participant testing protocols.

Hypotheses 1 and 2 were assessed using a mixed ANOVA, targeting the between-subjects effect of device and within-subjects interactions and pairwise comparisons of frequency*device and electrode*device. As multivariate analysis of variance (MANOVA) is not dependent upon the assumptions of sphericity, the Wilks’ Lambda multivariate test statistics are reported when applicable. Hypothesis 3 was assessed by calculating the bivariate correlation coefficient between the dry and wet electrode system for each frequency band during the eyes-closed and eyes-open in vivo recording protocol. As the directional nature of the correlations was hypothesized, one-tailed probabilities were reported for all correlations.

## Results

### Participants

A convenience sample of nine right-handed healthy adults (3 female, 6 male), ages 18–64 years (*M =* 47.11, *SD =* 15.19), volunteered to participate in the in vivo protocol of the current investigation.

### Experiment 1

**Fig. 4:**
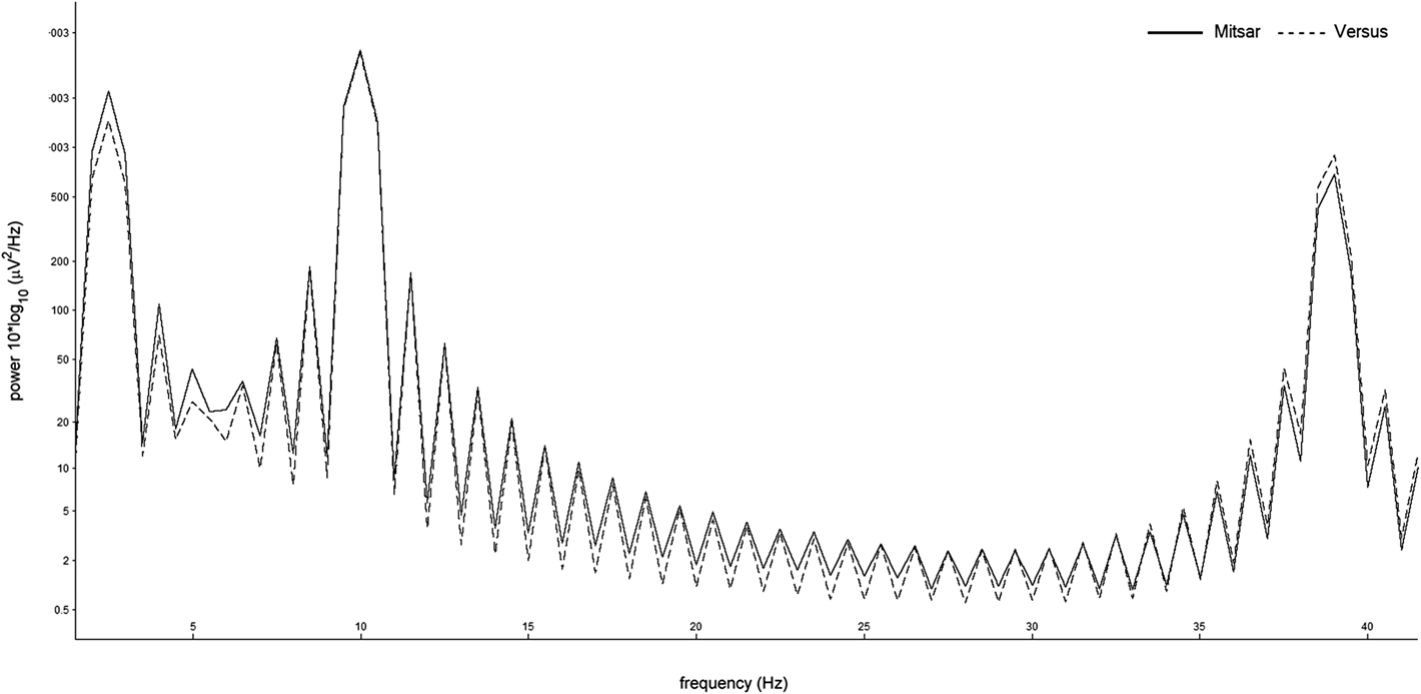
Grand-average power spectral density of signal generator test protocol. Solid line indicates the wet electrode system [Mitsar] output, the dashed lines indicate the dry electrode system [Versus] output. *Note.* Test signals: 2.5 Hz, 10 Hz, 39 Hz

In the analysis of the signal generator protocol, the between-subjects effect of device produced a non-significant main effect, *F*(1, 2) = 4.155, *p* = .178, *η*_*ρ*_^2^ = .675. Due to insufficient residual degrees of freedom, only the multivariate test statistic for the frequency*device interaction could be produced, indicating a non-significant interaction effect, Wilks' λ = .004, *F*(1, 2) - 114.366, *p* = .066, *η*_*ρ*_^2^ = .996. Figure 4 displays the grand-average power spectral density plot of signal generator test protocol.

### Experiment 2

**Fig. 5:**
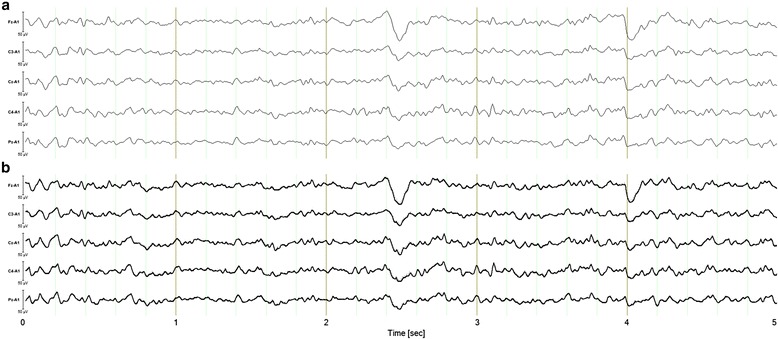
Representative five-second sample of simultaneous EEG traces taken from pairs of dry/wet electrode combinations for a single participant. **a** Upper panel indicates wet electrode system signal [Mitsar] output. **b** Lower panel indicates dry electrode system signal [Versus] output. *Note.* Sampling rate: 128 Hz, Filters: bandpass [1.5 Hz - 45 Hz] Gain: 50 uV

**Fig. 6:**
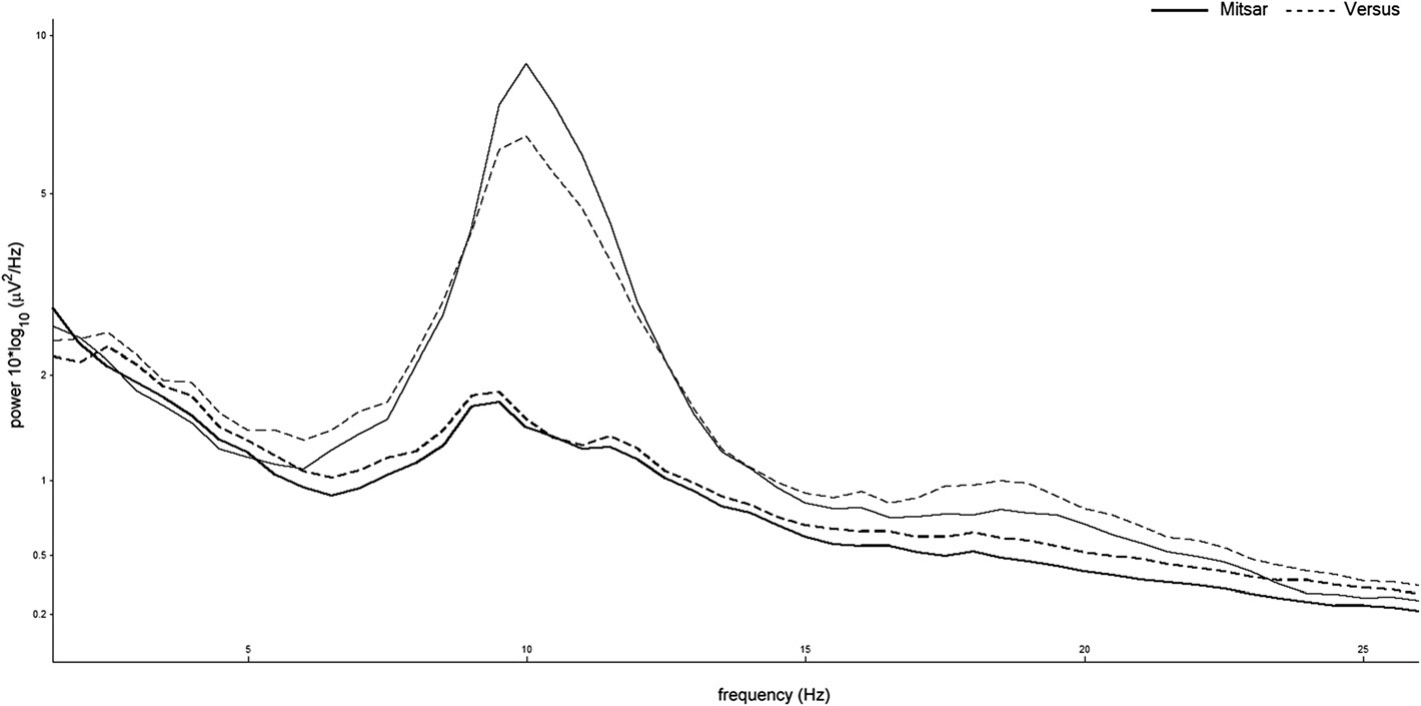
Grand-average power spectral density showing alpha wave attenuation at Pz during the eyes-open and eyes-closed recording conditions. Bold tracings indicate the eyes-open condition, lighter tracings indicate the eyes-closed condition. Solid line indicates the wet electrode system [Mitsar] output, the dashed lines indicate the dry electrode system [Versus] output

In the analysis of the in vivo participant protocol, the between-subjects effect of device produced a non-significant main effect for the eyes-closed recordings, *F*(1, 16) = .338, *p* = .569, *η*_*ρ*_^2^ = .021, and the eyes-open recordings, *F*(1, 16) = .061, *p* = .808, *η*_*ρ*_^2^ = .004. The multivariate test statistic for the frequency*device, Wilks' λ = .682, *F*(6, 11) = .856, *p* = .554, *η*_*ρ*_^2^ = .318, and electrode*device, Wilks' λ = .701, *F*(4, 13) = 1.388, *p* = .292, *η*_*ρ*_^2^ = .299, revealed non-significant interaction effects in the eyes-closed data. Similarly, the multivariate test statistic for the frequency*device, Wilks' λ = .835, *F*(6, 11) = .362, *p* = .888, *η*_*ρ*_^2^ = .165, and electrode*device, Wilks' λ = .629, *F*(4, 13) = 1.918, *p* = .167, *η*_*ρ*_^2^ = .371, for the eyes-open data revealed non-significant interaction effects. Figure 5 provides a five-second sample of eyes-open simultaneous EEG traces taken from both dry/wet electrode systems for a single participant. Figure 6 provides the grand-average power spectral density plots for the participant sample, showing alpha wave attenuation at Pz during the eyes-open and eyes-closed recording conditions.

**Fig. 7:**
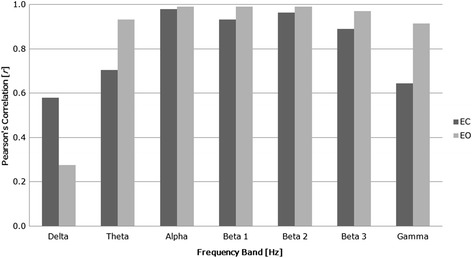
Average correlations for each frequency band. Average correlations of dry and wet electrode signals across recording condition and electrode site for each frequency band [delta (1.5-3.5 Hz), theta (4–7.5 Hz), alpha (8–12 Hz), beta 1 (13–16 Hz), beta 2 (13–21 Hz), beta 3 (21–32 Hz), gamma (35–45 Hz)]

**Fig. 8:**
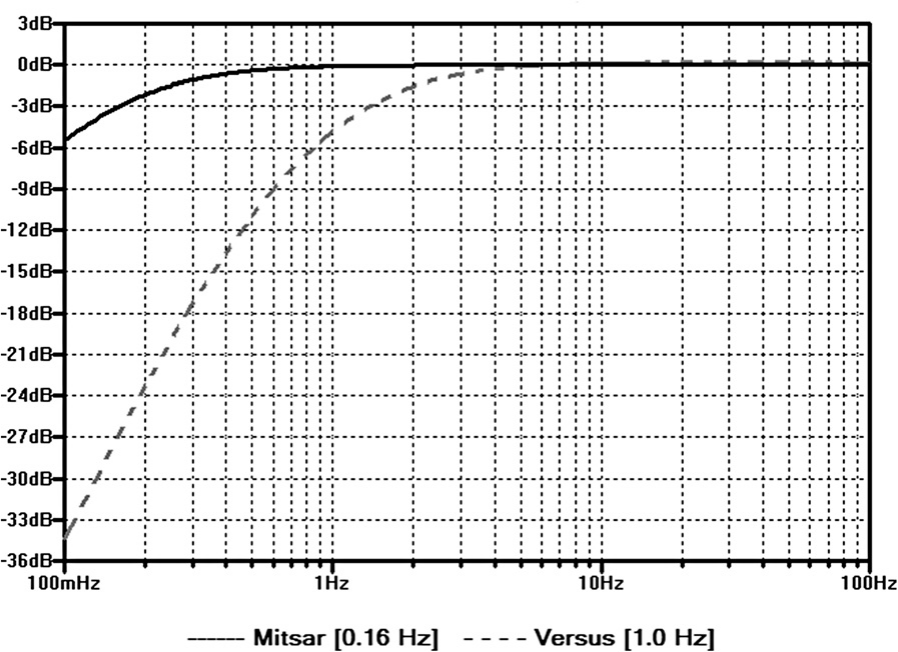
Comparison of frequency response using different high-pass filters. Solid line indicates the wet electrode system [Mitsar] frequency response with 0.16 Hz high-pass filter, dashed line indicated the dry electrode system [Versus] frequency response with 1.0 Hz high-pass filter

In the correlation analysis, a significant positive relationship (all *p*'s < .05) between the dry and wet electrode signal amplitude was observed for theta, alpha, beta 1, beta 2, beta 3, and gamma in both the eyes-closed and eyes-open conditions. Additionally, large correlation coefficients (*r* > *±* 0.5) were observed for these measures. A large correlation coefficient was observed for eyes-closed delta activity, *r* = .580, *p* = .051, and a small correlation coefficient was observed for eyes-open delta activity, *r* = .278, *p* = .236, however, these positive relationships did not reach the level of significance. Table 2 provides a numeric summary of the Pearson's *r* and significance values. Correlations of dry and wet electrode frequency activity averaged across electrode sites for each recording condition are displayed in Fig. 8.

**Table 2:** Pearson’s(r) correlations values for dry and wet electrodes

Variables	EC		EO	
	*r*	*p*	*r*	*p*
Delta	0.58	.051	0.28	.236
Theta	0.70	.017*	0.93	.000**
Alpha	0.98	.000**	0.99	.000**
Beta 1	0.93	.000**	0.99	.000**
Beta 2	0.96	.000**	0.99	.000**
Beta 3	0.89	.001**	0.97	.000**
Gamma	0.64	.031*	0.91	.000**

Note. Pearson's correlation coefficient effects sizes, ±.1 represents a small effect, ±.3 represents a medium effect, and ± .5 represents a large effect, EO = eyes-open, EC = eyes-closed. *Correlation significant at 0.05 level (1-tailed), **Correlation significant at 0.01 level (1-tailed)

## Discussion

In this study, serial simulated and simultaneous in vivo evaluation methods were employed to compare the signal output of EEG data recorded from a wireless dry electrode system (Versus) and a conventional wet electrode system (Mitsar-201). electrodes. Hypothesis 1 was confirmed, as no between-group effects for device and no frequency*device or electrode*device interaction effects were observed in delta, alpha, or gamma amplitudes for the comparison of signal generated activity at 2.5 Hz, 10 Hz, and 39 Hz. Hypothesis 2 was also confirmed, as no between-group effects for device and no frequency*device or electrode*device interaction effects were observed in delta, theta, alpha, beta 1, beta 2, beta 3, or gamma amplitudes for the comparison of simultaneous eyes-closed and eyes-open recordings in a sample of healthy adults. Hypothesis 3 was partially confirmed, as 85 % of the signal output correlations between devices produced a significant positive relationship (*p* > .05) under the eyes-closed and eyes-open conditions (Fig. 7), with 92 % demonstrating a large effect size (*r* > .5). Non-significant correlations and reduced effect sizes where observed in the delta frequency band, and predominantly during the eyes-open recording condition.

**Fig. 9:**
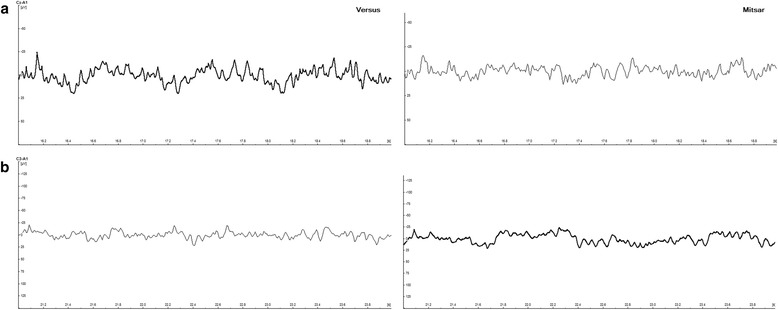
Slow-wave artifact patterns. Three-second sample of central slow-wave artifacts observed during the eyes-open simultaneous EEG recordings of two participants, left panel = Versus dry electrode, right panel = Mitsar wet electrode. (a) Upper panel = increased slow -wave activity observed on Versus dry electrode [bold], (b) Lower panel = increased slow -wave activity observed on Mitsar wet electrode [bold], *Note.* Sampling rate: 128 Hz, Filters: bandpass [1.5 Hz - 45 Hz] Gain: 50 uV

Several factors may account for the diminished correlation coefficients and effect sizes observed in the delta band activity. Factor 1, differences in delta activity may have been the product of differences in electrode placement rather than electrode detection. Lopez-Gordo and colleagues [6] assert that using a "same-time-different-place" approach for validation testing is controversial, as electrodes placed in different locations measure different ionic currents and different electrical activity. For the simultaneous recordings, the Mitsar wet electrodes for Fz and Cz were placed slightly off the midline towards the left hemisphere, while the electrodes corresponding to C3, C4, and Pz were placed to the posterior of the dry electrodes. However, difference in electrode position would likely be consistent across all frequency bands and both conditions. Factor 2, differences in delta activity may have been the product of differences in hardware based filters for both devices. Gargiulo and colleagues [6] suggest that the frequency bandwidth of a reference device and device under test should be identical, but warn researchers that this is not always possible due to non-excludable hardware and notch filtering. Although the raw data was exported and analyzed in a third-party software, the Versus dry electrode system utilizes a non-excludable high-pass filter of 1Hz at the hardware level, while the Mitsar wet electrode system utilizes a high-pass filter of 0.16 Hz. Fig. 8 provides a comparison of the frequency response of each device using different high-pass filters. It can easily be observed that the 1.0 Hz high-pass filter of the dry electrode system diminishes the delta frequency response. This would likely produce the largest amplitude differences during the eyes-open condition, as blinks and eye-movement are more prominent and produce large slow wave amplitudes in the delta frequency range. Factor 3, differences in delta activity may have been the product of individual participant artifacts during the recording. Review of the eyes-open raw recordings revealed two participants with increased slowing on one or more of the central electrode sites; one with prominent artifacts on the wet electrode system and one with prominent artifacts on dry electrode system (Fig. 9). Exclusion of these participants' central delta activity in the eyes-open condition increased the mean correlation coefficient, *r* = .541, *p* = .066, producing a large effect size and similar values observed in the eye-closed condition.

Limitations are present in the current research design, including a small sample size, limited number of comparative reference sites, limited study tasks and environments, and reference device differences. Future investigations should address the study limitations by (1) recruiting a larger sample, (2) employing parallel and serial comparison methods or additional electrodes to the anterior and posterior position of the dry electrodes to generate an averaged comparison signal, (3) assessing event related potentials and/or include tasks designed to elicit a variety of mood and performance states, (4) EEG assessment in non-laboratory settings, (5) investigations of controlled physiological artifacts including electromyography and electrooculargraphy, (6) development of bypass or excludable filters for reference device testing, and (7) further characterization of the electrical circuit and signal response. Despite the current limitations, this investigation has many strengths, including the use of simulated, in vivo, and comparative evaluations techniques with multiple frequency ranges, recording conditions, and a reference device, qualitative physiological signal evaluation at the individual and group level, and quantitative evaluation of the device characteristics.

## Conclusions

The present study provides preliminary data pertaining to the validity of a specific wireless headset with dry electrodes. Overall, the data suggest that the raw EEG data recorded by the wireless dry electrode system is of adequate quality to that of conventional wet electrode EEG systems. These results are promising, as wireless dry electrode technology has several advantages over conventional systems. These include increased portability (wireless, rechargeable), ease of use and decreased setup times for clinicians, participants, and researchers (self-application, paste/gel free, 2-min setup), reduced equipment costs (dry system $400, conventional system $10,500), and the opportunity to unobtrusively assess or train EEG activity at 5 standard electrode sites (Fz, C3, Cz, C4, Pz) in a variety of settings and tasks - enhancing the ecological validity.

## Abbreviations

EEG: Electroencephalogram
FFT: Fast Fourier Transform
